# Pre-obese children’s dysbiotic gut microbiome and unhealthy diets may predict the development of obesity

**DOI:** 10.1038/s42003-018-0221-5

**Published:** 2018-12-07

**Authors:** Simone Rampelli, Kathrin Guenther, Silvia Turroni, Maike Wolters, Toomas Veidebaum, Yiannis Kourides, Dénes Molnár, Lauren Lissner, Alfonso Benitez-Paez, Yolanda Sanz, Arno Fraterman, Nathalie Michels, Patrizia Brigidi, Marco Candela, Wolfgang Ahrens

**Affiliations:** 1 Microbial Ecology of Health Unit, Department of Pharmacy and Biotechnology, University of Bologna, Via Belmeloro 6, 40126 Bologna, Italy; 2 Leibniz Institute for Prevention Research and Epidemiology—BIPS, Achterstraße 30, 28359 Bremen, Germany; 3 Department of Chronic Diseases, National Institute for Health Development, Hiiu 42, 11619 Tallinn, Estonia; 4 Research and Education Institute of Child Health, Stavrou Street 56, 2035 Strovolos, Cyprus; 5 Department of Paediatrics, Clinical Center, University of Pécs, József Attila u. 7, 7623 Pecs, Hungary; 6 Section for Epidemiology and Social Medicine, University of Gothenburg, Medicinaregatan 16, 40530 Gothenburg, Sweden; 7 Microbial Ecology, Nutrition & Health Research Unit, Institute of Agrochemistry and Food Technology, National Research Council (IATA-CSIC), Avda. Catedrático Agustín Escardino 7, 46980 Valencia, Spain; 8 Medizinisches Versorgungszentrum Dr. Eberhard & Partner Dortmund, Laboratoriumsmedizin Dortmund, Brauhausstraße 4, 44137 Dortmund, Germany; 9 Department of Public Health, Ghent University, Corneel Heymanslaan 10, 9000 Ghent, Belgium

**Keywords:** Microbiome, Obesity

## Abstract

It is widely accepted that the intestinal microbiome is connected to obesity, as key mediator of the diet impact on the host metabolic and immunological status. To investigate whether the individual gut microbiome has a potential in predicting the onset and progression of diseases, here we characterized the faecal microbiota of 70 children in a two-time point prospective study, within a four-year window. All children had normal weight at the beginning of this study, but 36 of them gained excessive weight at the subsequent check-up. Microbiome data were analysed together with the hosts’ diet information, physical activity, and inflammatory parameters. We find that the gut microbiota structures were stratified into a discrete number of groups, characterized by different biodiversity that correlates with inflammatory markers and dietary habits, regardless of age, gender, and body weight. Collectively, our data underscore the importance of the microbiome–host–diet configuration as a possible predictor of obesity.

## Abstract (web summary)

Simone Rampelli, Kathrin Guenther, and their colleagues discover correlations between pre-obese children’s low-diverse, dysbiotic microbiome configurations and their unhealthy diets. This study suggests that diet interventions may promote healthy adulthood by modulating the intestinal microbiota.

## Introduction

Obesity and associated metabolic diseases are linked to diet and gut microbiome in an intimate way^1^. Prevalence rates of obesity have increased dramatically in the past decades. In 2014, 1.9 billion adults worldwide were overweight and 600 million of them were obese (World Health Organization, 2016, http://www.who.int/mediacentre/factsheets/fs311/en/). Obesity, deriving from a positive energy balance that results from a surplus in ingested with respect to the expended energy, is considered a major risk factor for health, with important consequences on quality of life, life expectancy, and healthcare costs^2^. The intestinal microbiome is a pivotal emerging factor that can affect human metabolic homeostasis and promote the risk of metabolic complications connected to obesity. Even if there is a lack of consensus on the obese-type microbiome configuration, taxonomic and functional alterations have been suggested to contribute to the pathogenesis of obesity in both humans and animal models^3–5^. The altered microbial profile occurring in obese people is considered as an extreme deviation from the microbiota–host mutualism, resulting from the response to a high-fat high-sugar diet^5^. The obesity-related gut microbiota is generally characterized by a low degree of biodiversity and enrichment in pathobiont bacteria, such as members of the family *Enterobacteriaceae*, as well as *Erysipelotrichaceae* and the sulphate reducer species *Bilophila wadsworthia*^4,6^. This dysbiotic microbial structure is probably involved in the manifestation of obesity in a multifactorial way^7^. Coherently with the energy harvest hypothesis^3^, the gut microbiome of obese individuals possesses higher efficiency in energy extraction from the diet, providing an extra supply of calories to the host^8–10^. Furthermore, the concomitant overload of the intestinal microbial ecosystem with pro-inflammatory *Enterobacteriaceae* and sulphate-reducing bacteria may consolidate the obesity-associated inflammation and insulin resistance^11^.

The prevalence of obesity is increasing worldwide, particularly, in children^12^, and this has been closely associated with cardiovascular risk factors, such as hypertension, insulin resistance, and dyslipidaemia, during adulthood^13^. However, links among diet, microbiome structure, and child health are still unclear. To test the hypothesis that the composition and/or the diversity of the microbiome had an impact on the onset of obesity, we explored the faecal microbiota structure in 70 children in a prospective study, at a baseline survey and a follow-up after 4 years. All children were normal weight at baseline, but 36 developed an excessive weight gain until follow-up. We also collected comprehensive data on lifestyle, such as dietary intake and physical activity, as well as medical history, anthropometry, measures of physiological, immunological, psychological parameters, and socioeconomic status. According to our findings, clusters of dietary patterns are associated with specific differences in the gut microbiota and health datasets: the normal weight children ate differently and had a distinctive microbiome configuration compared to obese children. Measures of increased inflammation in obese children suggest that dietary adjustments might promote healthier adolescence and adulthood by modulating the intestinal microbiota.

## Results

### Microbiota structure, health correlations, and healthy growth

To investigate links between the gut microbiota and obesity, health, diet, and other lifestyle factors, we analysed the faecal microbial composition in 70 children at two time points (T1, T3) within a 4-year window. Children were stratified by timing and weight status: at T1, all children were normal weight of which 34 are referred as T1_N who remained normal weight (mean ± sd, age (years): 7 ± 2; BMI (kg m^−2^): 16 ± 1), and 36 as T1_O (age: 8 ± 2; BMI: 16 ± 2), who gained excessive weight; accordingly, at T3, 34 subjects had maintained their normal weight (T3_N; age: 11 ± 2; BMI: 17 ± 2), while 36 had gained excessive weight (T3_O; age: 12 ± 2; BMI: 20 ± 3).

We generated 7.9 million sequence reads from 16s rRNA gene V3–V4 amplicons, with an average of 56,485 (±22,321, sd) paired-end reads per sample, for 20,360 OTUs (Operational Taxonomic Units) grouped at 97% of sequence identity. When examining OTU abundance, we identified four subject clusters, one of which (C3) included the majority of obese subjects, before and after they had developed obesity (Supplementary Fig. 1). For 18 out of the 70 children, the most relevant variable that drove the separation was the sample origin, with samples T1 and T3 from the same individual clustering together in the dendrogram (*p* = 0.0001, Fisher’s test).

**Fig. 1:**
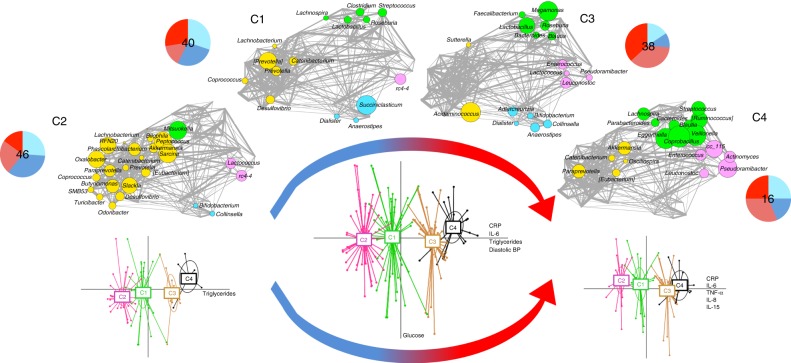
Variation of the gut microbiota structure across normal weight and obese children is mirrored by changes in health indices. The PCoA plots (PCo1 and PCo3 axes used) in the lower part of the figure show four significantly different groups of subjects (C1–C4, *p* < 0.001), as defined by unweighted UniFrac microbiota analysis of normal weight children (T1_N, T3_N; left), the whole cohort (centre) and obese children (T1_O, T3_O; right). The top of the figure shows the Wiggum plots corresponding to the four groups from the whole cohort analysis, in which disc sizes indicate genus over-abundance compared to the average relative abundance in the whole cohort. Pie charts show the proportion and number of subjects per group (pink, T1 normal weight children that will develop obesity (T1_O); red, T3 obese children (T3_O); cyan, T1 normal weight children (T1_N); light blue, T3 normal weight children (T3_N)). For subject clustering (C1–C4), please see Supplementary Fig. 1. Curved arrows indicate a transition from health (blue) to an inflammatory state (red), as defined by the increase in several inflammatory markers (CRP, IL-6, IL-8, IL-15, TNF-α), as well as in triglycerides and diastolic blood pressure. Please see also Table 1

To identify trends in the gut microbiota, we established co-abundance associations of genera (Supplementary Fig. 2), and then clustered correlated bacterial taxa into four co-abundance groups (CAGs), describing the microbiota structures found across the whole dataset. The dominant (i.e. the most abundant) genera in these CAGs were *Bacteroides* (green), *Prevotella* (yellow), *Dorea* (violet), and *Bifidobacterium* (blue). The CAG relationships are termed Wiggum plots, where genus abundance is represented as a disc proportional to normalized over-abundance (Fig. 1). The four subject divisions, as identified by OTU clustering (Supplementary Fig. 1), were superimposed on the unweighted (Fig. 1) and weighted (Supplementary Fig. 3) UniFrac Principal Coordinates Analysis (PCoA) plots, allowing defining four clusters, C1–C4. Within a spectrum of microbiota structures, these clusters represent groups of individuals who have a significantly different microbiota layout from each other, as defined by the permutation multivariate analysis of variance (MANOVA) test on UniFrac data (*p* < 0.001). We then constructed Wiggum plots for the gut microbiota for each of the 4 groups (Fig. 1). The microbiota variation from the groups dominated by normal weight children (C1/C2) to the groups dominated by obese children (C3/C4) was accompanied by distinctive CAG dominance, most relevantly by abundances of *Prevotella* CAG (C1) and *Dorea* and *Bacteroides* CAGs (C4). Significant associations between several health/inflammation measurements and the major axes from unweighted UniFrac PCoA analysis are shown in Table 1. In particular, when considering the whole cohort, a shift of the microbiota structure towards positive values of the PCo1 axis was associated with inflammation, i.e. serum levels of C-reactive protein (CRP) and IL-6. Interestingly, other inflammatory markers, such as interleukin-15 (IL-15), tumour necrosis factor alpha (TNF-α), interferon gamma-induced protein 10 (IP-10), interleukin 6 (IL-6), and interleukin 8 (IL-8), correlated only with the microbiota profiles from children developing obesity. As expected, there was minimal variability amongst normal weight subjects. It should be noted that the education level score and physical activity score (time spent in moderate to vigorous physical activity, MVPA) were also associated with the microbiota structure, but in an independent way with respect to inflammatory parameters and the lean/obese phenotype (Supplementary Fig. 4). Furthermore, the microbial biodiversity was associated with the inflammatory status. Indeed, we observed a gradual change of the level of biodiversity along PCo1, from the highest level in the samples belonging to the C2 cluster to the lowest values in the C4 microbiomes (*p* < 0.000001, Kruskal–Wallis test; Supplementary Fig. 5). On the other hand, when comparing the biodiversity of the child microbiota among the original groupings (T1_N, T3_N, T1_O, T3_O), we only detected a significant difference using the Shannon index. In particular, we found higher biodiversity in the T1_N microbiome than T1_O samples (*p* < 0.01, Wilcoxon test), but this evidence was not confirmed with the other metrics.

**Table 1:** Regression tests of associations between clinical measurements and microbiota composition

Parameter	PCo1			PCo2			PCo3		
	RC range	RC sd	*p*	RC range	RC sd	*p*	RC range	RC sd	*p*
**(a) Unweighted UniFrac PCoA for all subjects**									
ISCED (education level score)	−0.95934	−0.15989	0.3	0.369546	0.10869	0.04	0.350038	0.13463	0.3
Evenson MVPA (moderate to vigorous physical activity) score	0.64632	0.10772	0.5	−0.415378	−0.12217	0.05	0.387634	0.14909	0.2
CRP	3.10902	0.51817	0.0003	0.000374	0.00011	0.9	−0.05109	−0.01965	0.9
Triglyceride	1.83048	0.30508	0.04	0.071706	0.02109	0.7	0.149942	0.05767	0.6
IL-6	2.34798	0.39133	0.03	0.082212	0.02418	0.8	−0.284232	−0.10932	0.5
Diastolic blood pressure	2.24544	0.37424	0.04	0.32045	0.09425	0.3	0.33124	0.1274	0.4
Glucose	0.2517	0.04195	0.8	0.131818	0.03877	0.7	−0.951886	−0.36611	0.02
**(b)** **Unweighted UniFrac PCoA for developing obesity-only subjects (T1_O, T3_O)**									
Evenson MVPA (moderate to vigorous physical activity) score	−0.23454	−0.03909	0.8	−0.440368	−0.12952	0.04	0.076778	0.02953	0.8
CRP	3.18672	0.53112	0.007	−0.097478	−0.02867	0.7	−0.327028	−0.12578	0.2
TNF-α	2.78556	0.46426	0.04	−0.09656	−0.0284	0.8	−0.691912	−0.26612	0.3
IP-10	2.99568	0.49928	0.05	0.250036	0.07354	0.4	−0.479206	−0.18431	0.4
IL-8	3.16782	0.52797	0.04	−0.031246	-0.00919	0.9	−0.313976	−0.12076	0.6
IL-6	3.00882	0.50147	0.04	0.181254	0.05331	0.6	−0.701142	−0.26967	0.3
IL-15	2.92746	0.48791	0.04	0.16286	0.0479	0.7	−0.434798	−0.16723	0.6
**(c)** **Unweighted UniFrac PCoA for normal weight-only subjects (T1_N, T3_N)**									
Weight	−0.81234	−0.13539	0.4	−0.646952	−0.19028	0.05	−0.317382	−0.12207	0.5
Triglyceride	1.94376	0.32396	0.04	0.462876	0.13614	0.2	0.41379	0.15915	0.4

Quantile (median) regression tests of associations between metadata measurements and microbiota composition as measured by unweighted UniFrac PCoA across all groups (all children, a; T1_O/T3_O-only children, b; T1_N/T3_N-only children, c). Column headings are: RC range, regression coefficients scaled to the full variation along each PCoA axis, thus indicating direction and magnitude of the association; RC sd, regression coefficients scaled to one standard deviation; *p*, quantile regression *p* value

It should be noted that both the unweighted and weighted UniFrac PCoA space was not correlated with either the child’s age (*p* = 0.7 for unweighted UniFrac; *p* = 0.6 for weighted UniFrac, permutational correlation test), gender (*p* = 0.2 for unweighted UniFrac; *p* = 0.4 for weighted UniFrac; permutational MANOVA) or maturation stage according to Tanner classification^14^ (*p* = 0.2 for unweighted UniFrac; *p* = 0.6 for weighted UniFrac), meaning that the associations described above were irrespective of these variables.

Taken together, these results indicate that the gut microbiota may exist under a number of configurations, which are associated with host metabolic and immunological factors and, in the context of other individual lifestyle and genetic variables, may be involved (or not) in the development of the multifactorial obese phenotype.

### Impact of diet on the gut microbiota

**Fig. 2:**
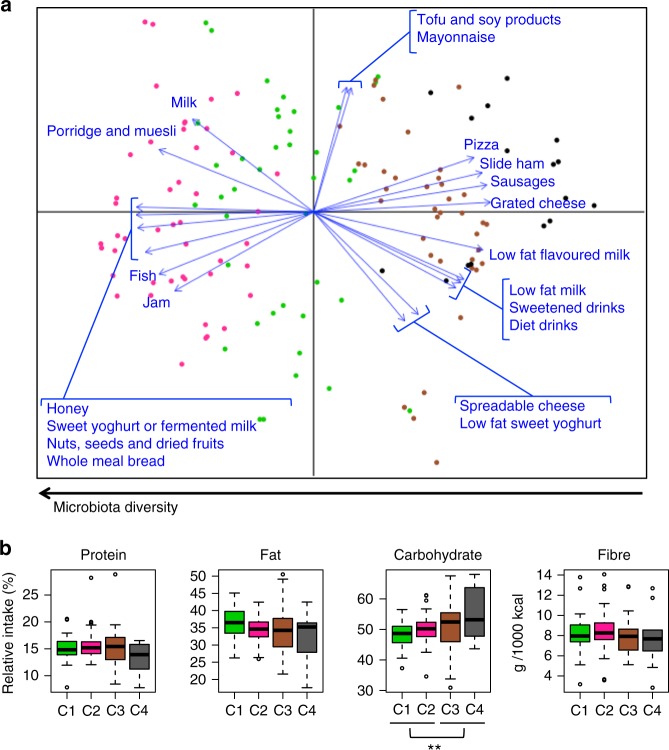
Differences in food consumption lead to different microbiota configurations. **a** PCoA based on unweighted UniFrac distances of the faecal microbiota of 70 children at two time points, as shown in the centre of Fig. 1. The biplot of the average food coordinates weighted by frequency of consumption per sample was superimposed on the PCoA plot to identify the foods contributing to the ordination space (blue arrows). Only the food categories showing a highly significant correlation with the sample separation (*p* < 0.005, permutational correlation test) were displayed. Samples are coloured by subject group (C1–C4), as in Fig. 1. The black arrow at the bottom indicates the direction of the microbiota diversity gradient along PCo1. **b** Summary of the macronutrient intake, expressed as a percentage of kilocalories consumed per day, and fibre consumption, as grams of fibre intake per 1000 kilocalories consumed. Data are presented for each of the four microbiota groups. *p* value < 0.05 was indicated in the figure (**)

To identify the food types with the most significant contribution (*p* < 0.05, permutational correlation test) to the microbiota ordination, we superimposed the food data from Food Frequency Questionnaires (FFQs) on the unweighted UniFrac PCoA plot of Fig. 1 (Fig. 2a). Remarkably, a higher consumption of milk, fish, seeds, and whole meal bread was associated with the configurations C1 and C2 of the gut microbiota. On the other hand, the microbiota configurations C3 and C4 were associated with a higher consumption of dairy products, pizza, sausages, and sweetened drinks. Differently, as mentioned above, the microbiota diversity was inversely correlated to the first axis. In line with the available literature on the diet as the major driver of the microbiota structure^15^, differences in food consumption may thus contribute to differences in microbiota diversity between groups. Mean values of food consumption per day for each of the four microbiota groups are reported in Supplementary Table 1, along with more information on food categories. Furthermore, by focusing on macronutrients, we found that the most discriminant category was carbohydrate, whose consumption increased in a gradual manner along the first axis (Fig. 2b). In light of the fact that diet, in terms of excess energy intake, is a major cause of obesity^1^, it is important to note that the microbiota configurations (C1–C4) were independent of the total daily caloric intake, and that an increase of caloric intake was observed at T3, compared to the first time point, in accordance with the growth of children (Supplementary Fig. 6).

### Microbiota, diet, and physical activity relation to obesity

**Fig. 3:**
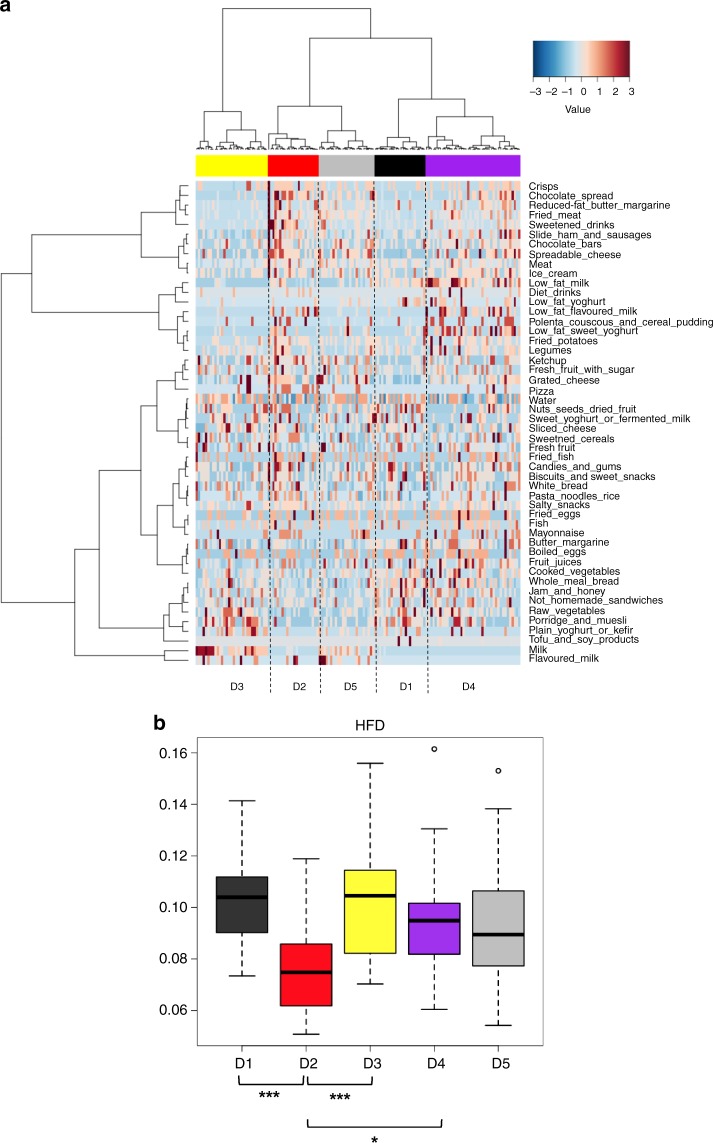
Dietary patterns discriminate children for the Healthy Food Diversity index. **a** Five dietary groups (D1–D5) revealed through Ward linkage clustering using Euclidean distances applied to the first eigenvector in a Correspondence Analysis of data from Food Frequency Questionnaires. **b** Comparison of the Healthy Food Diversity (HFD) index^16^ across the five dietary groups identified in **a**. *p* values < 0.0001 (***) and <0.01 (*) were indicated in the figure

FFQ data were further explored in a Correspondence Analysis, where the first axis, describing over 9% of the dataset variance, contained most of the discriminating food types identified in the previous correlation analysis of FFQ data on the microbiota PCoA, such as milk, pizza, and sweetened drinks. Application of Ward linkage clustering and Euclidean distance metrics to this axis allowed identifying five dietary groups (*p* < 0.001, Fisher’s test): D1 (‘low protein/low carbohydrate’), D2 (‘high carbohydrate/high fat’), D3 (‘high carbohydrate/high fibre’), D4 (‘low protein/low fat’), and D5 (‘high protein/high fat’) (Fig. 3a). We then calculated the Healthy Food Diversity (HFD) index, an index used to measure dietary diversity, based on evidence that a diverse diet promotes health status^16^. By analysing samples by dietary groups rather than microbiota groups, we found out that the diet was the least diverse in D2, while showing the highest diversity in D1 and D3 (*p* = 0.0002, Kruskal–Wallis test; Fig. 3b). When we focused on the sample distribution in a longitudinal way, we observed that for 16 out of 70 children, the T1 and T3 samples fell in the same dietary group. Twelve children changed their diet group from D1 to D3 or vice versa, thus maintaining a high HFD index. Only four children (2 T1_O/T3_O and 2 T1_N/T3_N) modified their dietary group in the worst way, i.e. from diets with the highest HFD index (D1, D3) to the least diverse diet, D2 (Supplementary Data 1).

By matching the stratifications of subjects in dietary and microbiota groups, we sought for redundant combinations associated with the obese phenotype. In particular, in the light of the obtained results, the combination D2 diet and C3/C4 microbiota was exclusively associated with a disease-promoting and inflammation status. Seven obese children out of 36 were D2–C3/C4, whereas none of T1_N/T3_N children possessed this configuration (*p* = 0.0006, Fisher’s exact test). It is important to note that the only T1_N child who fell in the D2 group (C2 microbiota group) showed a high MVPA score (higher than 75% of T1 subjects), suggesting a protective role of physical activity in children consuming a diet associated with a low HFD index. This was also confirmed for the combination D5 diet and C3/C4 microbiota, in which fell seven obese children and only one T1_N child with high MVPA score (*p* = 0.008). However, it should be pointed out that, even if the combinations D2/D5 diet and C3/C4 microbiota were associated with the obese phenotype, there were obese children harbouring different profiles. This stresses that obesity is a complex mosaic, in which several endogenous and exogenous factors, including host genetics, contribute to health decline. Interestingly, when looking at T1 samples in a prospective manner, we consolidated our hypothesis, by detecting D2/D5–C3/C4 configurations exclusively in normal weight children who showed excessive weight gain at T3. This finding suggests a sort of predictive potential of the microbiome–host–diet configuration, even if the model clearly needs to be implemented with more subjects, sampling points and other omics data to increase statistical power. The assignment of the dietary group, microbiota group, and physical activity score is reported in Supplementary Data 1 for each sample.

### Microbiota signatures of obesity

**Fig. 4:**
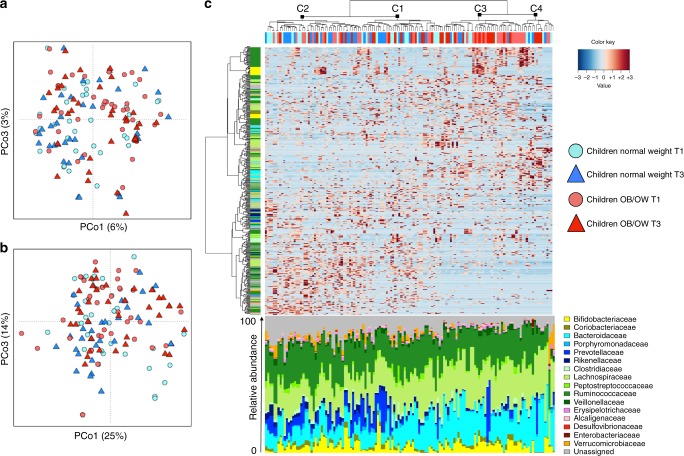
Microbiota analysis separates children based on obesity. **a** Unweighted and **b** weighted UniFrac PCoA of the faecal microbiota from 70 children, at two different time points. Subject colour coding: pink, T1 normal weight children that will develop obesity; red, T3 obese children; cyan, T1 normal weight children; light blue, T3 normal weight children. **c** Hierarchical Ward linkage clustering based on the Spearman correlation coefficients of the relative abundance of OTUs, filtered for OTU presence in at least 20% of the subjects. Subject colour coding as in **a**. Labelled groups in the top tree (basis for the four groups in Fig. 1) are highlighted by black squares. OTUs are colour-coded by family assignment in the vertical tree. Bacteroidetes phylum, blue gradient; Firmicutes, green; Proteobacteria, red; and Actinobacteria, yellow. Four hundred fifty-six OTUs classified to the family level are visualized. The bar plot shows the relative abundance of the family-classified microbiota profiles

UniFrac PCoA analysis showed weak but significant separation between subjects with (T3_O) or without obesity (T1_N, T3_N, T1_O), according to both unweighted (*p* = 0.02, permutation test with pseudo-*F* ratios; Fig. 4a) and weighted (*p* = 0.05; Fig. 4b) distance metrics. Family-level microbiota assignment highlighted a readjustment within the phylum Bacteroidetes, with a higher proportion of *Bacteroidaceae* and a lower proportion of *Prevotellaceae* in obese children when compared to the normal weight counterparts (Fig. 4c). In addition, obese children showed a higher contribution of the genus *Lachnospira* compared to normal weight children at the same time point (T3_N). When looking at T1_O/T3_O children in a longitudinal way, we denoted an increase in the relative abundance of Proteobacteria and a decrease in the proportions of the families *Clostridiaceae* and *Ruminococcaceae* after the onset of obesity. On the other hand, when focusing on normal weight children, we observed only a sensible reduction in Proteobacteria at the second time point (T3_N) compared to the baseline. A complete summary of the significant differences in the gut microbiota between groups is reported in Table 2. It is important to note that differences between obese (T3_O) and non-obese (T1_O and T3_N) children involved major microbiota components, whereas differences between non-obese children (T1_N, T3_N, and T1_O) involved only minor components, proving that obesity is associated with certain gut microbiota profiles, although alone cannot be used as unique predictive tool.

**Table 2:** Microbial taxa significantly different across the four groups of children

Differences between normal weight children at T1						
Taxon	Level	Mean T1_N	SEM T1_N	Mean T1_O	SEM T1_O	*p*
Cyanobacteria	Phylum	0.19	0.07	0.14	0.07	0.05
*S24-7*	Family	0.7	0.25	0.11	0.08	0.05
*Slackia*	Genus	0.05	0.01	0.03	0.01	0.008
*[Prevotella]*	Genus	0.3	0.18	0	0	0.05
*Lactococcus*	Genus	0.12	0.04	0.1	0.07	0.04
**Differences between normal weight children at T1 and T3**						
**Taxon**	**Level**	**Mean T1_N**	**SEM T1_N**	**Mean T3_N**	**SEM T3_N**	***p***
Proteobacteria	Phylum	1.51	0.19	1.08	0.11	0.04
Tenericutes	Phylum	0.6	0.18	0.18	0.08	0.03
*[Mogibacteriaceae]*	Family	0.14	0.02	0.18	0.02	0.03
*Enterobacteriaceae*	Family	0.32	0.17	0.04	0.02	0.01
*Catenibacterium*	Genus	0.07	0.04	0.23	0.1	0.04
**Differences between children before and after the onset of obesity**						
**Taxon**	**Level**	**Mean T1_O**	**SEM T1_O**	**Mean T3_O**	**SEM T3_O**	***p***
Proteobacteria	Phylum	1.1	0.13	1.56	0.24	0.02
*Lactobacillaceae*	Family	0.2	0.13	0.11	0.07	0.02
*Clostridiaceae*	Family	1.5	0.2	1.14	0.17	0.03
*Ruminococcaceae*	Family	24.82	1.16	21.55	0.98	0.02
*Alcaligenaceae*	Family	0.66	0.09	0.86	0.09	0.03
*Ruminococcus*	Genus	5.92	0.86	3.88	0.47	0.05
*Sutterella*	Genus	0.66	0.09	0.86	0.09	0.03
**Differences between normal weight and obese children at T3**						
**Taxon**	**Level**	**Mean T3_N**	**SEM T3_N**	**Mean T3_O**	**SEM T3_O**	***p***
*Bacteroidaceae*	Family	15.76	1.86	19.62	1.68	0.03
*Prevotellaceae*	Family	6.16	1.45	5.23	1.74	0.02
*Christensenellaceae*	Family	0.49	0.11	0.29	0.10	0.05
*[Mogibacteriaceae]*	Family	0.18	0.02	0.11	0.02	0.001
*[Tissierellaceae]*	Family	0.02	0	0.01	0	0.04
*[Cerasicoccaceae]*	Family	0.10	0.06	0	0	0.002
*Slackia*	Genus	0.05	0.01	0.02	0.01	0.008
*Bacteroides*	Genus	15.76	1.86	19.62	1.68	0.03
*Prevotella*	Genus	6.16	1.45	5.23	1.74	0.02
*[Prevotella]*	Genus	0.26	0.12	0.03	0.03	0.02
*Lachnospira*	Genus	0.74	0.10	1.44	0.21	0.02
*Roseburia*	Genus	0.13	0.02	0.23	0.04	0.03
*Oscillospira*	Genus	0.80	0.05	0.66	0.05	0.04

Only taxa found in at least 20% of the samples were considered. Differences in relative abundance were assessed by Wilcoxon test, paired or unpaired as needed

## Discussion

In this study, we explored the structure of the gut microbiota of 70 children in a two-time point prospective study, with samplings being separated by a period of 4 years. During this time window, half of the children maintained their normal weight, while the rest gained excessive weight. The data were integrated with dietary intake information, measures of physical activity and inflammatory parameters. By analysing the degree of similarity among the 140 gut microbial profiles, we identified four significantly different compositional clusters, each representing a gut microbiota steady state. For each of these steady states, we obtained a peculiar compositional profile, as highlighted by the respective dominance of the four CAGs. In particular, the steady state C1 was characterized by *Prevotella* and *Bacteroides* CAGs, while the steady state C2 was dominated by the *Prevotella* CAG. Conversely, steady states C3 and C4 were more heterogeneous, with the first showing the concomitant presence of all the four CAGs (*Prevotella*, *Bacteroides*, *Bifidobacterium*, and *Dorea*), and the second lacking only the *Bifidobacterium* CAG. Steady states were characterized by a different degree of bacterial diversity, with C2 showing the highest level of microbial diversity followed by C1, C3, and C4. When seeking for the prevalence of sample types within the four steady states, we found that samples from pre-obese (T1_O) and obese (T3_O) children were largely more prevalent in the low-diversity clusters, C3 and C4. In addition, when we explored connections between the gut microbiome steady states and inflammation, we found positive associations with C3 and C4. Taken together, our data indicate that the low-diversity gut microbiota configurations C3 and C4 can represent obesogenic gut microbiome layouts, predisposing children to metabolic inflammation and obesity.

In light of the importance of diet as a gut microbiome modulator^15,17–23^, we explored connections between dietary habits and microbiome steady states. To this aim, the individual dietary profiles at T1 and T3 were clustered into five dietary groups, from D1 to D5, each being characterized by a different abundance of macronutrients: protein, fat, and carbohydrate. When we calculated the HFD index^16^, the dietary clusters D1 and D3 showed the highest potential to promote health, while D2 the lowest. Interestingly, when we sought for redundant combinations between diet and microbiome clusters, potentially predisposing to obesity, we found that the combinations D2/D5 and C3/C4 were more prevalent in pre-obese (T1_O) and obese children (T3_O) compared to the other subjects. In particular, the combination D2 diet and C3/C4 microbiome steady state was exclusively observed in obese children, while seven of the eight children that showed the combination D5 and C3/C4 were obese. Finally, the combinations D2/D5 and C3/C4 at T1 were distinctive of normal weight children who showed excessive weight gain at T3, supporting the relevance of the combination of diet and microbiome structure as a possible predictor of obesity. It is also tempting to speculate that the differences in food intake contributed to the observed microbiota differences. Consistent with the gut microbiome arrangements reported in other studies, we observed higher levels of *Bilophila* in children that consumed more milk^24^, and higher contribution of *Prevotella* in children with higher intake of whole meal bread^25^. On the other hand, we found more *Bacteroides* and *Oscillospira* in children who ate more ham and sausages, as already described in adults following an animal-based, low-fibre diet^15^. As demonstrated by Zhernakova and colleagues^26^, we also found evidence of an inverse correlation between microbiota diversity and consumption of sugar-sweetened drinks. It is worth noting that a high diversity in the gut microbial ecosystem, together with high levels of short-chain fatty acid production were reported in rural children of Burkina Faso, whose diet is rich in complex carbohydrates and fibre^27^. Similarly, a high-diversity gut microbiome, with enrichment of genes involved in the metabolism of complex polysaccharides, was found in the Hadza, a hunter-gatherer population following a heavily plant-based diet^28^. In line with these findings, our results showed that the microbiota diversity was higher in children who ate more foods containing oligosaccharides, such as honey and whole milk^29^, with the latter being also a source of fat-soluble Vitamin D, whose deficiency is associated with obesity in children and adolescents^30^. The microbiota diversity was also higher in children with high consumption of complex polysaccharides, such as whole meal bread, nuts, and seeds. The link between diet and microbiota also clearly involves human physiology. Indeed, it has been demonstrated that the dietary fat increases the amount of endotoxins in the blood^31^, and that circulating endotoxin levels are associated with elevated TNF-α, IL-8, and IL-6 concentrations^32,33^. In agreement with these data, we found higher plasmatic levels of IL-6, IL-8, and TNF-α associated with an overabundance of gram-negative bacteria, such as *Veillonella*, *Akkermansia*, *Bacteroides*, and *Parabacteroides*, in the C3/C4 configurations. On the other hand, we found that the consumption of fish was directly connected to a microbiota configuration with low inflammatory grade, as it has been reported for lard-consuming mice transplanted with the microbiota of fish oil-consuming mice^34^. Importantly, the health–microbiota associations were statistically significant even when the model was adjusted for age, and robust to gender and maturation stage according to Tanner classification^14^.

Finally, we also detected robust microbiome signatures of obesity as previously reported^1,4,6^, such as the reduction of the ecosystem diversity. Furthermore, a higher proportion of *Bacteroidaceae* and a lower abundance of *Prevotellaceae* characterized the obese children with respect to normal weight children, consistently with the long-term dietary effects reported by Wu and colleagues^17^. Looking longitudinally, the onset of obesity in children was connected to an increase of Proteobacteria and a decrease of *Clostridiaceae* and *Ruminococcaceae*, suggesting that the obesity development also involves changes in the abundance of key gut microbiome components.

**Fig. 5:**
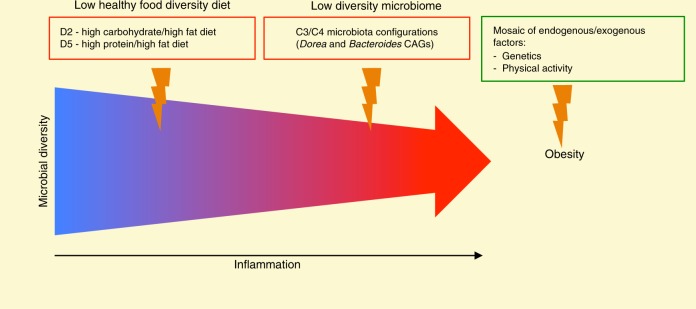
The mosaic aetiology of obesity. The gut microbiota diversity is likely altered at multiple stages by the diet. Unhealthy diets may promote an inflammatory state that, in turn, is strictly interconnected with the gut microbial configuration. The combination of these three factors (unhealthy diets, inflammation and a dysbiotic, low-diverse and pro-inflammatory microbial layout) may favour the onset of obesity. High physical activity may protect the human host from obesity, even when diet and microbiota are in a low-diversity and pro-inflammatory configuration. However, human genetics can lead the host to develop obesity, regardless of the microbiome configuration

In conclusion, our data highlight the importance of the individual microbiome configuration as a mediator of the dietary impact on the individual metabolic and immunological homeostasis. According to our findings, the individual gut microbiome configuration—in terms of steady state—together with the long-term dietary habit can be considered as a predictive tool for the development of obesity in children (Fig. 5). Hence, our data pave the way for a new perspective, where dietary recommendations to reduce the obesity risk in children are specifically tailored based on the individual microbiome structure, with the precise purpose of avoiding combinations of diet and microbiome configuration that are likely to favour the onset of obesity. Our data also stress the multifactorial nature of obesity, where gut microbiome dysbioses and interacting factors (e.g. diet) are only a part of the complex mosaic of determinants of this phenotypic trait (Fig. 5). Future studies on larger cohorts, based on shotgun metagenomics and possibly providing for more extensive sampling, are needed to better unravel the contribution of the gut microbiota, as well as of specific species and/or strains, to this complex mosaic.

## Methods

### The IDEFICS/I.Family cohort

The sample comprised children derived from the surveys of the ‘Identification and Prevention of Dietary- and Lifestyle-Induced Health Effects in Children and Infants’ (IDEFICS) cohort study and from the project ‘Investigating the determinants of food choice, lifestyle and health in European children, adolescents and their parents’ (I.Family). The IDEFICS study is a prospective cohort of 16,228 children aged 2–9 years from eight European countries (Belgium, Cyprus, Estonia, Germany, Hungary, Italy, Spain, and Sweden), from kindergartens and schools. The IDEFICS study consisted of one baseline survey (T0) performed from September 2007 to May 2008 and one follow-up survey (T1), which was conducted 2 years later (September 2009 to July 2010). The surveys provided information about dietary habits, physical activity, sociodemographic factors, clinical and physical examinations, and health outcomes. The follow-up project I.Family represents an extension of the IDEFICS study (T3) and was conducted in 2013–2014, in which children who participated in T0 and/or T1 were followed up for the third time complemented with information from their parents and siblings. Details of the design and methods of these surveys have been described elsewhere^35^.

The present study is based on a subgroup of IDEFICS/I.Family children who provided stool samples. The first stool samples were collected in 2010 during the second survey of IDEFICS (T1) in five of the eight participating countries (Cyprus, Estonia, Germany, Hungary, and Sweden). A second stool sample was collected in these countries during the follow-up at T3. None of the children took antibiotics in the 14 days before sample collection. All applicable institutional and governmental regulations concerning the ethical use of human volunteers were followed during this research. Approval by the appropriate ethics committees (Cyprus National Bioethics Committee, Cyprus, 12/Jul/2007, No. EEBK/EM/2007/16 and 21/Feb/2013, No. EEBK/ETI/2012/33; Tallinn Medical Research Ethics Committee (TMREC), Estonia, 14/Jun/2007, No. 1093 and 17/Jan/2013, No. 128; Ethic Commission of the University of Bremen, Germany, 16/Jan/2007 and 11/Dec/2012; Medical Research Council, Hungary, 21/Jun/2007, 22-156/2007-1018EKU and 18/Dec/2012, 4536/2013/EKU; Regional Ethics Research Board in Gothenburg, Sweden, 30/Jul/2007, No. 264-07 and 10/Jan/2013, No. 927-12) was obtained by each of the centres doing the fieldwork. The parents or guardians as well as children from the age of 12 years gave their written informed consent and younger children expressed their oral consent for the examinations and data collection.

### Sample collection

The IDEFICS and I.Family examinations of children included the collection of biological samples (blood, urine, saliva, faeces). Venous blood was drawn after an overnight fast using standardized procedures^36^ by all survey centres, and stored at −80 °C. Faeces were collected in a subsample with the PSP® Spin Stool DNA PLUS Kit (Stratec Molecular, Berlin, Germany) at home. The stool collection kit included a collection tube with a DNA stabiliser, an illustrated description of how to collect the stool samples, a short questionnaire and a paper stool collector. The participant had to collect one spoon of the middle of the faecal sample and to mix the sample by shaking. The samples were stored at −20 °C on the day of collection and then transferred to −80 °C upon arrival in the laboratory. More details about children are reported in Supplementary Data 1.

### Collection of clinical, behavioural, and nutritional data

Examinations of children included anthropometry, blood pressure, accelerometry, genetic data from saliva, and physiological markers in blood and urine. Supplementary Table 2 gives an overview on the assessment methods applied in the study and a description of the collected variables.

Dietary intake and behaviour were measured in detail using a validated semi-quantitative FFQ^37,38^ and a self-administered computer-assisted 24-h dietary recall, which is linked to a tailor-made European database of food composition tables^39,40^ as described below.

### DNA extraction and sequencing

Total microbial DNA was extracted from faecal samples by the repeated bead-beating plus column method^41^ with some additional steps as reported by Turroni et al.^42^. The V3–V4 hypervariable region of the 16s rRNA gene was amplified using the 341F and 805R primers with added Illumina adapter overhang sequences as previously reported^43^. Indexed libraries were prepared by limited-cycle PCR using Nextera technology and the final library was diluted to 6 pM with 20% PhiX control. Sequencing was performed on Illumina MiSeq platform using a 2 × 300 bp paired-end protocol, according to the manufacturer’s instructions.

### Bioinformatics and biostatistics

Paired-end reads were processed combining PANDAseq^44^ and QIIME^45^. High-quality sequences were clustered into OTUs at 97% sequence similarity by UCLUST^46^. Taxonomy was assigned with the RDP classifier against the Greengenes database (May 2013 release). Chimeric OTUs were identified using ChimeraSlayer^47^ and then filtered out. All singleton OTUs were discarded.

Alpha-diversity was evaluated using three different metrics: Shannon, Phylogenetic Diversity (PD) whole tree, and observed OTUs. Weighted and unweighted UniFrac distances were used to perform PCoA. PCoA, heatmap, and bar plots were built using the packages Made4^48^ and Vegan (http://www.cran.r-project.org/package=vegan).

Steady states were identified through hierarchical Ward linkage clustering based on the Spearman correlation coefficients of the proportion of OTUs, filtered for OTU subject prevalence of at least 20%. We then verified that each cluster showed significant correlations between samples within the group (multiple testing using the Benjamini–Hochberg method) and that the clusters were statistically significantly different from each other (permutational MANOVA using the Spearman distance matrix as input, function Adonis of the vegan package in R).

The R packages Stats and Vegan were used for statistical analysis. In particular, to compare the gut microbiota structure among different groups for α-diversity and macronutrient intake, we used the Wilcoxon test. The significance of data separation in the PCoA was assessed by a permutation test with pseudo-*F* ratios (function Adonis in Vegan). Cluster separation in hierarchical clustering analyses was tested using Fisher’s exact test. Significant differences in bacterial relative abundance at different phylogenetic levels among groups were assessed by Mann–Whitney *U* tests or Kruskal–Wallis test. *p* values were corrected for multiple comparisons using the Benjamini–Hochberg method when appropriate. False discovery rate (FDR) ≤ 0.05 was considered as statistically significant.

### Correlation analysis of clinical data and gut microbiota

Correlations between microbiota composition and host metadata, including inflammatory markers and other health parameters were analysed using quantile (median) regression tests, adjusted for age. Median regression is less influenced by outliers than the classical linear regression because it gives less relevance to extreme values. The potential impact of gender and maturation stage according to Tanner classification^14^ (whose information was available only at T3 for children who were 8 years old or older, i.e. 68 out of 70) on the microbiota structure was also evaluated. We carried out the analysis by using the R package quantreg, as already performed by Claesson et al.^49^.

### Analysis of nutritional data

Dietary data was collected through a semi-quantitative FFQ, weighted by 7-day consumption frequencies. Forty-six items were in common between FFQs at T1 and T3. Additional four items were obtained from questions about the type of milk and yoghurt consumed (skimmed or full-fat). For all FFQs the lowest frequency option was ‘never or less than once a week’, for foods with the highest frequency, the option was ‘4 or more times per day’^37,38^. At the same time, subjects were asked to compile 24-h dietary recalls with their parents, for retrieving more detailed information about the composition of their diet^39,40^. After considering several methodological approaches to quantify food frequency, we elected to convert the frequency of consumption assessed with the FFQ to a continuous scale of daily consumption (e.g. if the food was eaten 2 times per day, then the daily consumption was 2). When the frequency was reported as a range (e.g. eaten 1–3 times per week), the mean of the range (e.g. 2) was used to calculate the daily consumption. The superimposition of the food frequencies on the microbiota PCoA space was conducted using the envfit function of the Vegan package of R. A corrected *p* value ≤ 0.01 was considered as statistically significant. Macronutrient data were taken from dietary recalls, in particular in I.Family (T3), dietary intake of the previous 24 h was assessed using the validated web-based SACANA (Self-Administered Children, Adolescents and Adult Nutrition Assessment) 24-h dietary recall tool, which is based on the validated SACINA (Self-Administered Children, Infants and Adult Nutrition Assessment) offline version^39^ used in the IDEFICS study (T1). Children reported their diet and entered the type and amount (g) of all drinks and foods consumed during the previous day. While in SACINA all information was reported by the parents, in SACANA, children reported for themselves with the help of their parents^50^ or from a dietician or trained study nurse during the survey examinations.

### Co-abundance analysis

CAGs were identified as previously described^49^. In brief, associations among bacterial genera, present in at least two samples with relative abundance >0.1%, were evaluated by the Kendall correlation test, displayed using hierarchical Ward clustering with the Spearman correlation-based distance metrics and utilized to determine co-abundant groups of bacterial genera. Significant associations were controlled for multiple testing using the *q*-value method (FDR ≤ 0.05)^51^. Permutational MANOVA was used to determine whether the CAGs were significantly different from each other. Wiggum plot networks were created using the Cytoscape software (http://www.cytoscape.org/), as previously reported^49^. The circle size represents the bacterial abundance and connections between nodes represent positive and significant Kendall correlations among genera (FDR ≤ 0.05).

## Electronic supplementary material

Supplementary Information

Supplementary Data 1

Description of Additional Supplementary Files

## Data Availability

Sequencing reads were deposited as raw data, as a whole and separately for each sample, along with available metadata, in the MG-RAST database with the accession codes mgm4780879.3 to mgm4781018.3 (https://www.mg-rast.org/mgmain.html?mgpage=project&project=mgp84098). All relevant data are available from the authors.
