# Supplementary material for: Pre-obese children’s dysbiotic gut microbiome and unhealthy diets may predict the development of obesity
Source: Commun Biol. 2018 Dec 7;1:222. doi: 10.1038/s42003-018-0221-5 (PMC6286349; doi:10.1038/s42003-018-0221-5)
Supplement: Supplementary file 1 — Supplementary Information [file 42003_2018_221_MOESM1_ESM.pdf]

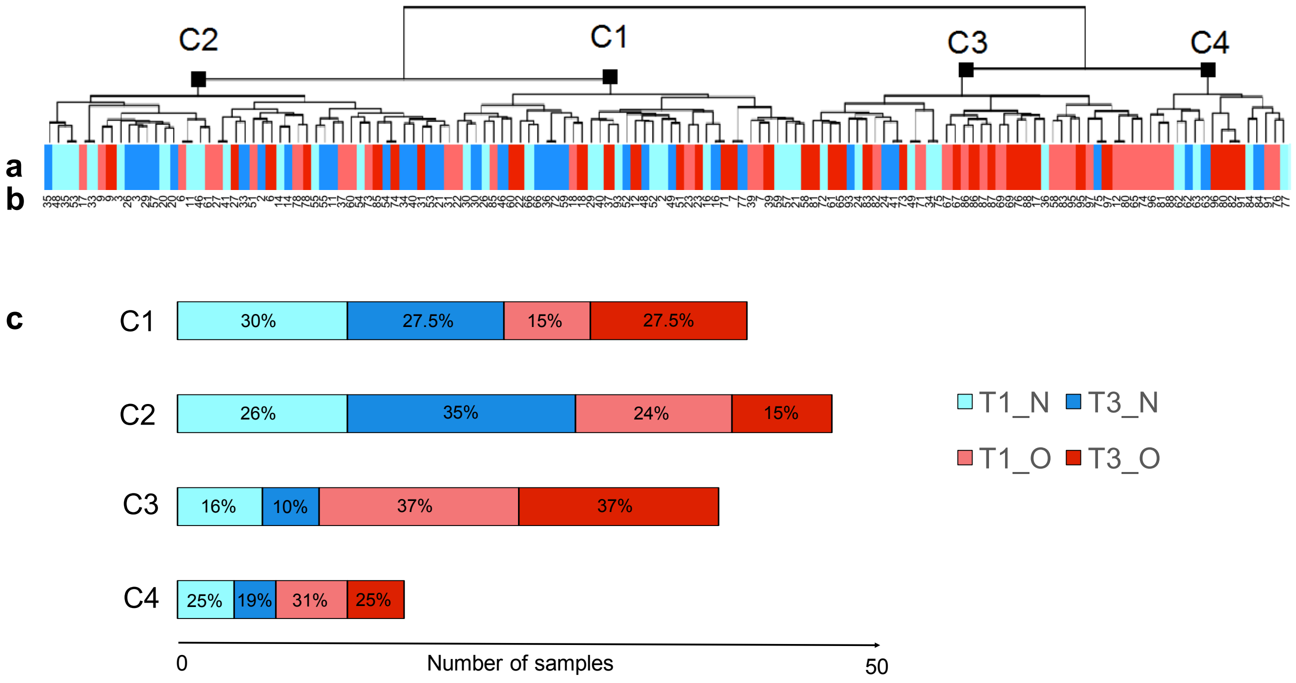

Supplementary Figure 1. Hierarchical Ward linkage clustering based on the Spearman correlation coefficients of the proportion of OTUs, filtered for OTU subject prevalence of at least 20%. Subject colour coding in **a:** pink, T1 normal weight children that will develop obesity (T1_O); red, T3 obese children (T3_O); cyan, T1 normal weight children (T1_N); light blue, T3 normal weight children (T3_N). Subject IDs are reported in **b**. The correspondence between subject IDs and sample IDs is shown in Supplementary Data 1. Labelled clusters in the top of the panel (representing the basis for the four groups in Fig. 1) are highlighted by black squares. The number and percentage of samples from T1_N, T3_N, T1_O and T3_O groups for each cluster are shown in **c** by bar plots.

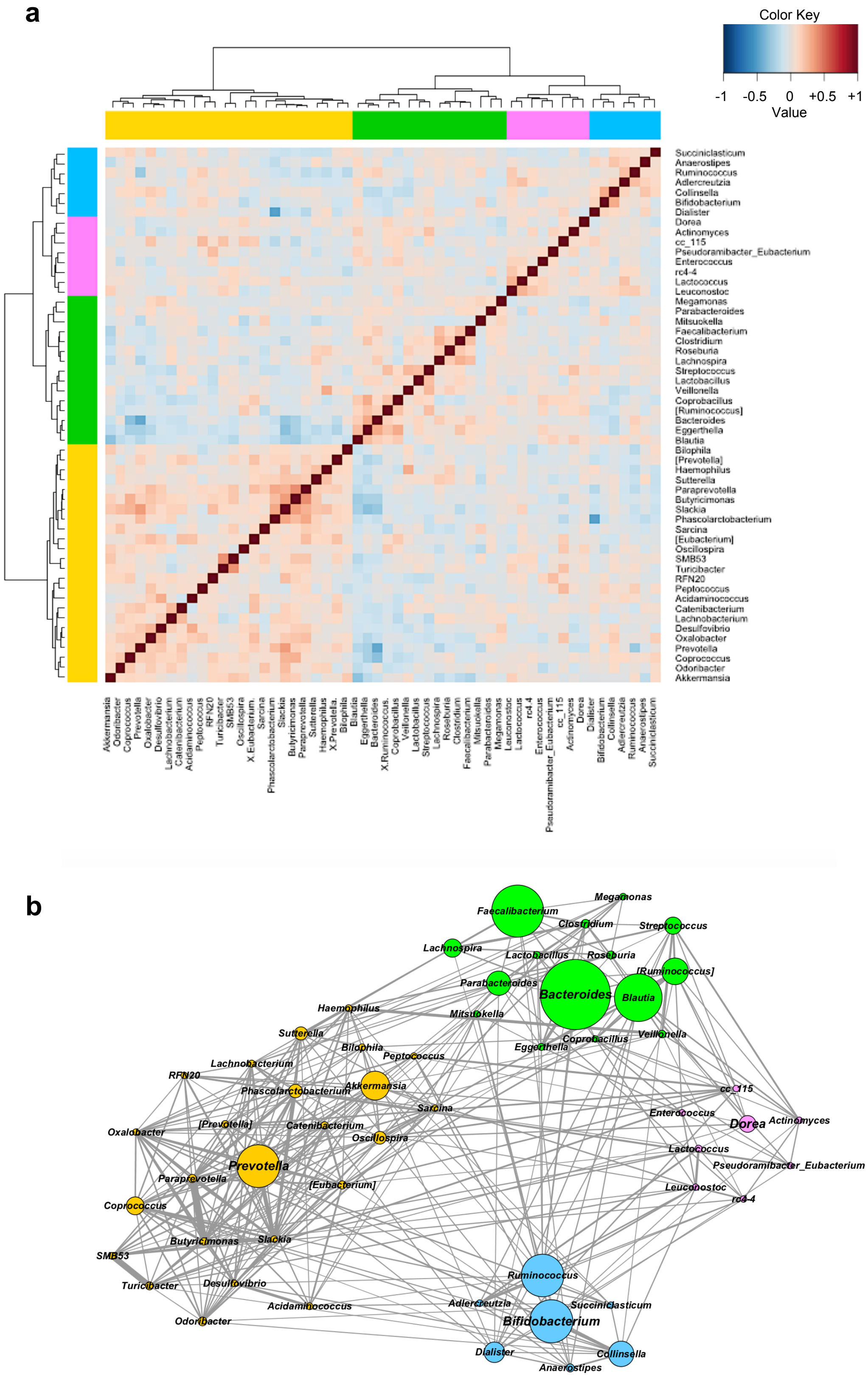

Supplementary Figure 2. Assignments of bacterial co-abundance groups (CAGs). CAG assignment relied on a heat plot (**a**) showing Kendall correlations between genera clustered by the

Spearman correlation coefficient and Ward linkage hierarchical clustering. Colours are indicative of the four identified CAGs. **b**, Wiggum plot correlations between the four CAGs identified. Circle size is proportional to the genus abundance and the connections between nodes represent positive and significant Kendall correlations between genera (FDR<0.05).

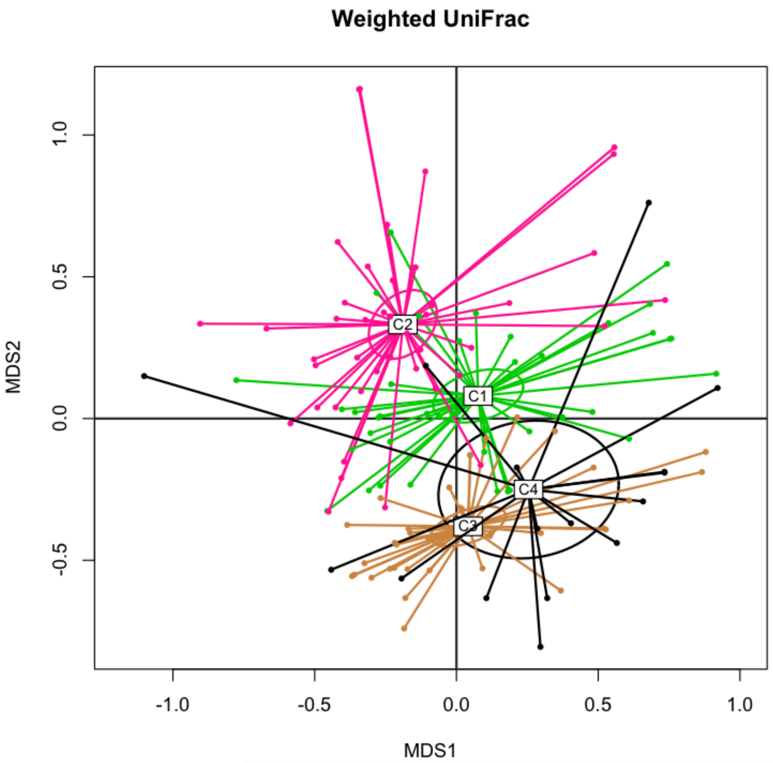

Supplementary Figure 3. Weighted UniFrac PCoA showing the four significantly different groups of subjects (C1 to C4, p<0.001, permutation test with pseudo-F ratios), as defined by OTU clustering (see Supplementary Figure 1).

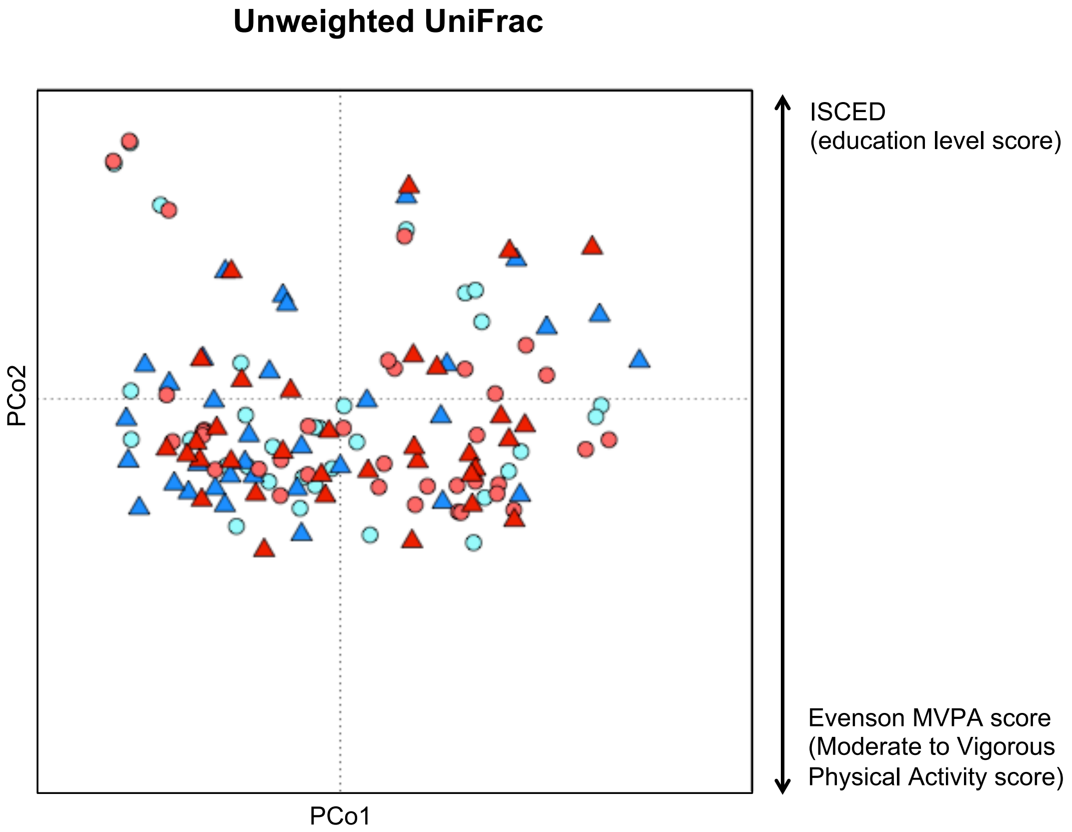

Supplementary Figure 4. Unweighted UniFrac PCoA of the faecal microbiota from 70 children at two different time points. Pink circles, T1 normal weight children that will develop obesity (T1_O); red triangles, T3 obese children (T3_O); cyan circles, T1 normal weight children (T1_N); light blue triangles, T3 normal weight children (T3_N). The variables significantly associated with the second axis are highlighted. See also Table 1.

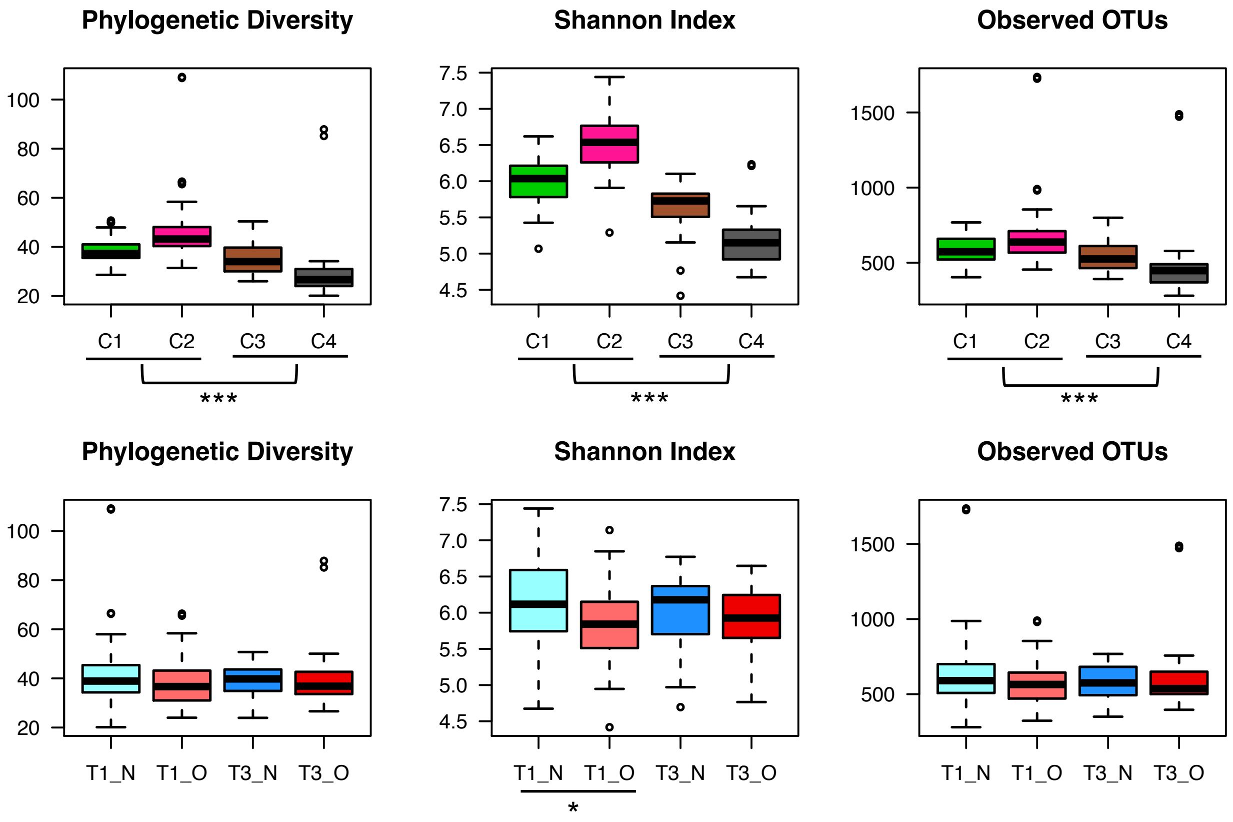

Supplementary Figure 5. Evaluation of α-diversity among sample groups. The OTU table at 0.03 similarity threshold was rarefied up to 12,000 reads per sample and analysed using various diversity metrics (Faith's Phylogenetic Diversity (PD whole tree), observed OTUs, and the Shannon index of biodiversity). According to all metrics, greater diversity (***, $p<0.000001$, Kruskal-Wallis test) was observed in C1/C2 compared to C3/C4 microbiota configuration (top). When clustered by T1_N, T1_O, T3_N and T3_O groups, we found a significant difference between T1_N and T1_O samples (*, $p<0.01$, Wilcoxon test) only when we used the Shannon index (bottom).

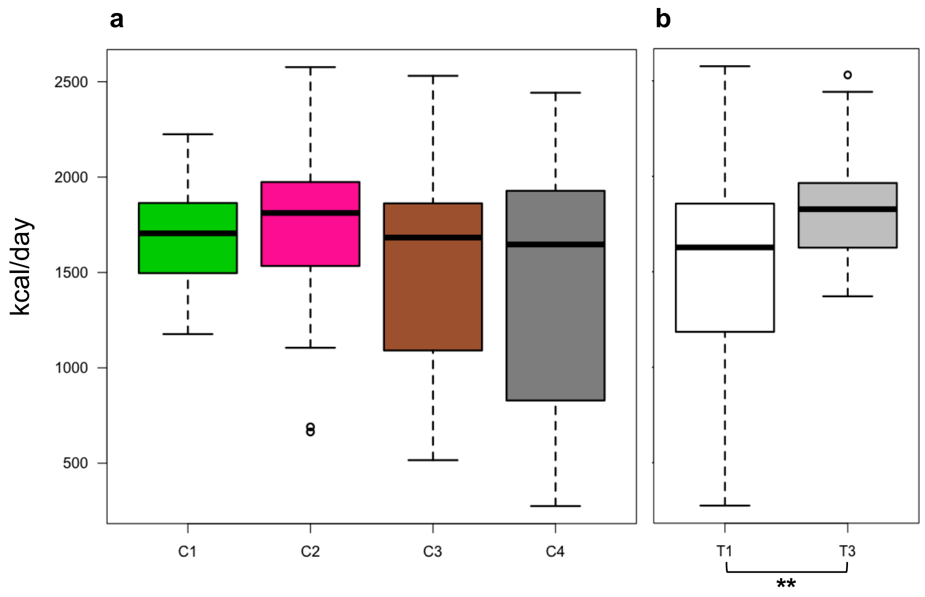

Supplementary Figure 6. Gut microbiota configurations and total daily caloric intake. **a**, Microbiota configurations (C1 to C4) are independent of the total daily kilocalorie intake. **b**, A significant increase in kilocalorie intake was observed at T3 compared to T1 (**, p<0.001, Wilcoxon test), in accordance with the growth of children.

Supplementary Table 1. Food description and average consumption values (grams per day) for each of the four microbiota groups

Food	Description	Food Category	Microbiota group			
			C1	C2	C3	C4
porridge and muesli	Porridge, oat meal, gruel, unsweetened cereals, plain muesli	Breakfast Cereals	0.298	0.365	0.202	0.200
sweetened cereals	sweetened or sugar added breakfast cereals and sweetened crisp muesli	Breakfast Cereals	0.464	0.462	0.366	0.325
not homemade sandwiches	Hamburger, hotdog, kebab, wrap, falafel	Cereal	0.260	0.308	0.240	0.181
pasta, noodles, rice	Pasta, noodles, rice	Cereal	0.368	0.410	0.400	0.544
Pizza	Pizza	Cereal	0.023	0.045	0.058	0.205
polenta, couscous and cereal pudding	Dish of milled cereals (rice or semolina pudding, polenta, couscous)	Cereal	0.061	0.051	0.051	0.078
white bread	White bread	Cereal	0.793	0.958	0.889	1.075
whole meal bread	Whole meal bread	Cereal	0.760	0.839	0.454	0.384
grated cheese	Grated cheese (parmesan, pecorino..)	Cheese	0.150	0.182	0.238	0.326
sliced cheese	Sliced cheese (Hard cheese like Chester, Emmental, Alpine cheese..)	Cheese	0.573	0.637	0.550	0.731
spreadable cheese	Spreadable cheese (brie, camembert and ricotta in addition to the cream cheese..)	Cheese	0.177	0.215	0.261	0.237
diet drinks	Diet and soft drinks	Drinks	0.015	0.028	0.173	0.000
fruit juices	Fruit Juices	Drinks	1.045	0.678	0.776	0.594
sweetened drinks	Sweetened drinks	Drinks	0.295	0.311	0.685	0.350
Water	Water	Drinks	2.010	2.350	3.089	3.456
boiled eggs	boiled or poached eggs	Eggs	0.168	0.153	0.101	0.156
fried eggs	fried or scrambled eggs	Eggs	0.198	0.150	0.105	0.158
Mayonnaise	Mayonnaise and mayonnaise based products	Eggs	0.078	0.089	0.084	0.144
Fish	Fresh or frozen or canned fish not fried	Fish	0.182	0.185	0.106	0.065
fried fish	Fried Fish	Fish	0.108	0.150	0.140	0.113
fresh fruit	Fresh fruit without added sugar	Fruit	0.725	0.933	1.179	1.338
fresh fruit with sugar	Fresh fruit with added sugar	Fruit	0.315	0.342	0.176	0.329
fried meat	fried meat	Meat	0.230	0.378	0.468	0.506
Meat	Fresh meat not fried	Meat	0.418	0.524	0.555	0.475
slide ham and sausages	Cold cuts and preserved, ready to cook meat product	Meat	0.470	0.474	0.750	0.869
tofu and soy products	Tofu, tempè, quorn, soy milk	Meat replacement product and soy product	0.008	0.000	0.000	0.019
flavoured milk	Sweetened or flavoured milk	Milk	0.065	0.081	0.064	0.063
Milk	Plain unsweetened milk	Milk	0.316	0.511	0.255	0.214
low fat milk	Sweetened or flavoured skimmed or low fat milk	Milk	0.707	0.541	0.718	0.909
low fat flavoured milk	Plain unsweetened skimmed or low fat milk	Milk	0.116	0.073	0.280	0.231
biscuits and sweet snacks	biscuits, packaged cakes or pastries	Snacks	0.240	0.324	0.341	0.131
candies and gums	Candies, loose candies and marshmallow	Snacks	0.290	0.312	0.307	0.144
chocolate bars	chocolate candy and bars	Snacks	0.271	0.279	0.267	0.256
Crisps	Crisps, corn crisps, popcorn	Snacks	0.105	0.165	0.191	0.181
ice cream	Ice cream, milk and fruit based bars	Snacks	0.215	0.244	0.277	0.110
nuts, seeds, dried fruit	Nuts, seeds and dried fruits	Snacks	0.172	0.295	0.144	0.138
salty snacks	Savoury pastries and fritters	Snacks	0.145	0.176	0.260	0.181
butter, margarine	Butter, margarine on bread	Spreadable products	0.815	0.765	0.932	0.688
chocolate spread	Chocolate or nut-based spread	Spreadable products	0.160	0.212	0.186	0.174
jam and honey	Jam, honey	Spreadable products	0.315	0.404	0.251	0.213
Ketchup	Ketchup	Spreadable products	0.250	0.313	0.297	0.337
reduced-fat butter, margarine	Reduced-fat butter or margarine	Spreadable products	0.111	0.169	0.236	0.426
cooked vegetables	Cooked Vegetable, potatoes and beans	Vegetable	0.741	0.607	0.592	0.663
fried potatoes	Fried potatoes	Vegetable	0.216	0.180	0.279	0.094

Food	Description	Food Category	Microbiota group			
			C1	C2	C3	C4
Legumes	Legumes	Vegetable	0.175	0.153	0.233	0.136
raw vegetables	raw vegetables (mixed salad, carrot, fennel, cucumber, lettuce, tomato)	Vegetable	0.720	0.752	0.576	0.900
plain yoghurt or kefir	Plain unsweetened yoghurt or kefir	Yoghurt	0.062	0.166	0.058	0.162
sweet yoghurt or fermented milk	Sweetened yoghurt or fermented milk	Yoghurt	0.221	0.300	0.113	0.131
low fat yoghurt	Plain unsweetened low fat yoghurt	Yoghurt	0.049	0.076	0.211	0.115
low fat sweet yoghurt	Sweetened low fat yoghurt	Yoghurt	0.126	0.124	0.242	0.113

Supplementary Table 2. Methods applied for anthropometric measurements and laboratory analyses

Measurement/ analysis	Device / Analysis method	Description	Reference
**Anthropometrics, blood pressure and physical activity**			
Height	Telescopic height measuring instrument (Seca 225 or 213 stadiometer, Birmingham, UK)	Measured barefoot to the nearest 0.1 cm.	S1
Weight	Electronic scale: Tanita BC 420 MA at T0/T1, for children ≤6 years adapted; BC 418 MA for children >6 years at T3 (Tanita Europe GmbH, Sindelfingen, Germany)	Children wore only underwear and a t-shirt, measured to the nearest 0.1 kg	S1
BMI z-score	Weight [kg] divided by height [m] squared	z-scores based on age- and sex-specific reference percentiles	S2
Blood pressure	Automated oscillometric device (Welch Allyn, Inc., 4200B-E2, Skaneateles Falls, NY, USA)	At the right arm after at least 5 minutes of rest in seated position. Two recordings with a 2-min interval, plus a third measurement in case of a >5% difference between the first two readings. Outcome: Mean value of the two measurements with the smallest difference	S3
Physical activity score (MVPA)	Uniaxial Actigraph accelerometer (Actigraph MTI, model GT1M, Manufacturing Technology Inc., Fort Walton Beach, FL, USA); ActiTrainer with the same technology as the Actigraph	Time spent in moderate-to-vigorous physical activity (MVPA) according to the cutpoints of Evenson et al. (2008)	S4, S5
**Biomarkers**			
Fasting glucose and triglycerides (TG)	Point-of-care analysis in one drop of native capillary or venous blood on the spot the Cholestech LDX analyzer (Cholestech, Hayward, CA) in T0/T1; Enzymatic colorimetric test (Cobas c701, Roche Diagnostics GmbH, Mannheim) in T3	The detection limit for TG was 0.509 mmol/l.	S6
C-reactive protein (CRP)	Serum high-sensitivity CRP concentrations measured with latex-enhanced nephelometry (BN2-Nephelometer, Siemens, Eschborn, Germany)	The serum CRP values were measured with a precision of 0.1 mg/l and a lower detection limit of 0.2 mg/l.	S7
TNF-α, IL-6, IL-8, IL-15, IP-10	Protein Multiplex analyzes with electrochemiluminescence technology from Meso Scale discovery (MSD); MULTI-SPOT ® Assay System; Cytokine Plate Human SP 6Plex: IL-6, IL-8, IL-15, IP-10, TNF-a, IL-1Ra	The detection limits observed with MSD MULTI-ARRAY and MULTISPOT Human Serum and Plasma Cytokine Assays are in the range of 0.5-10 pg/mL	

Supplementary References

S1. Stomfai, S. et al. Intra- and inter-observer reliability in anthropometric measurements in children. *Int. J. Obes. (Lond.)* **35**, S45-S51 (2011).

S2. Cole, T. J. & Lobstein, T. Extended international (IOTF) body mass index cut-offs for thinness, overweight and obesity. *Pediatr. Obes.* **7**, 284-294 (2012).

S3. Wolters, M. et al. Associations of whole blood n-3 and n-6 polyunsaturated fatty acids with blood pressure in children and adolescents - results from the IDEFICS/I.Family cohort. *PLoS One* **11**, e0165981 (2016).

S4. Evenson, K. R. et al. Calibration of two objective measures of physical activity for children. *J. Sports Sci.* **26**, 1557-1565 (2008).

S5. Konstabel, K. et al. Objectively measured physical activity in European children: the IDEFICS study. *Int. J. Obes. (Lond.)* **38**, S135-S143 (2014).

S6. Peplies, J. et al. Percentiles of fasting serum insulin, glucose, HbA1c and HOMA-IR in pre-pubertal normal weight European children from the IDEFICS cohort. *Int. J. Obes. (Lond.)* **38**, S39-S47 (2014).

S7. Schlenz, H. et al. C-reactive protein reference percentiles among pre-adolescent children in Europe based on the IDEFICS study population. *Int. J. Obes. (Lond.)* **38**, S26-S31 (2014).
