# Supplementary material for: Pre-obese children’s dysbiotic gut microbiome and unhealthy diets may predict the development of obesity
Source: Commun Biol. 2018 Dec 7;1:222. doi: 10.1038/s42003-018-0221-5 (PMC6286349; doi:10.1038/s42003-018-0221-5)
Supplement: Supplementary file 3 — Description of Additional Supplementary Files [file 42003_2018_221_MOESM3_ESM.docx]

**Description of Additional Supplementary File**

**Supplementary Data 1:** Summary of dietary group, microbiota group and physical activity score for each of the 70 subjects at two sampling time points
